# Oil palm kernel globulin antihypertensive peptides: isolation and characterization, ACE inhibition mechanisms, zinc-chelating activity, security and stability

**DOI:** 10.3389/fphar.2023.1225256

**Published:** 2023-08-02

**Authors:** Liangliang Zhang, Ding Pan, Lihua Shao, Yajun Zheng, Wenhui Hao, Yu Kan, Jiawei Cao, Haotong Yu, Jing Liu

**Affiliations:** Food Science College of Shanxi Normal University, Taiyuan, China

**Keywords:** oil palm kernel peptides, ACE inhibitory mechanism, zinc chelation, in silico screening, molecular docking, security, stability, spontaneous hypertensive rats

## Abstract

**Introduction:** The oil palm kernel (OPK) expeller is the main byproduct of palm oil, but its utilization is limited.

**Methods:** To obtain angiotensin-I-converting enzyme (ACE) inhibition peptides with Zn-chelating capacity, defatted oil palm kernel globulin hydrolysates (DOPKGH) were subjected to Sephadex G-15 gel electrophoresis, reverse-phase high liquid performance chromatography, and UPLC-ESI-MS/MS analysis.

**Results and discussion:** Five representative oligopeptides, including Gln-Arg-Leu-Asp-Arg-Cys-Lys (QRLERCK), Leu-Leu-Leu-Gly-Val-Ala-Asn-Tyr-Arg (LLLGVANYR), Arg-Ala-Asp-Val-Phe-Asn-Pro-Arg (RADVFNPR), Arg-Val-Ile-Lys-Tyr-Asn-Gly-Gly-Gly-Ser-Gly (RVIKYNGGGSG), and Glu-Val-Pro-Gln-Ala-Tyr-Ile-Pro (EVPQAYIP), without potential toxicity and allergenicity, were identified in DOPKGH. Of these, only EVPQAYIP showed both ACE-inhibitory activity (IC_50_: 102.75 μmol/L) and Zn-chelating capacity (11.69 mg/g). Molecular docking and inhibition kinetics showed that EVPQAYIP was a competitive inhibitor of ACE because it could bind to Glu384, Lys511, and Gln281 (belonging to the central S1 and S2 pockets, respectively) of ACE. Moreover, EVPQAYIP affects zinc tetrahedral coordination in ACE by binding to Glu411; the amino and carboxyl groups of EVPQAYIP chelate with zinc ions. During gastrointestinal digestion, the ACE inhibitory activity of EVPQAYIP was relatively stable. Additionally, EVPQAYIP enhanced zinc stability in the intestine and exerted antihypertensive effects in spontaneous hypertensive rats. These results suggest the potential application of OPK peptides as ingredients in antihypertensive agents or zinc fortification.

## 1 Introduction

An increasing number of studies have demonstrated the key role of angiotensin-I-converting enzyme (ACE) in blood pressure evaluation (Zhang et al., 2022). Thus, ACE is a primary target of antihypertensive drugs. ACE contains three active site pockets including S1, S2, and S′. Moreover, zinc tetrahedral coordination occurs in the catalytic center of ACE, consisting of a zinc ion bound to three ACE residues (Glu411, His387, and His383) (Fan and Wu, 2021). Previous studies have shown that agents can exert high ACE-inhibitory activity by binding to these active pockets, and that peptides with zinc-chelating ability can inhibit ACE by affecting zinc tetrahedral coordination (Wu et al., 2017; Li et al., 2022). In recent decades, ACE-inhibiting peptides derived from food have been extensively studied for their potential antihypertensive properties, affordability, and low risk of side effects (Lee and Hur, 2017). However, few studies have investigated the effects of peptides on ACE structure, especially on zinc tetrahedral coordination (Urbizo-Reyes et al., 2022).

Approximately one-sixth of the global population is zinc deficient (Sun et al., 2021). Zinc deficiency mainly causes anorexia, slow growth, low immune function, and cognitive impairment (Khan et al., 2022). Recently, food-derived peptides have been shown to be more effective, safe, and economical carriers for improving zinc absorption than inorganic zinc supplements such as zinc chloride and zinc sulfate (Ke et al., 2022). In the stomach and small intestine, zinc ions are protected by peptides against interference from phytic acid, fiber, and other agents (Zhang et al., 2018). In addition, zinc ions carried by peptides are mainly absorbed through oligopeptide absorption pathways, such as the peptide transporter I channel, bypass absorption, and endocytosis, which are faster and more stable than the ZIP and ZT systems (Udechukwu et al., 2018). Therefore, food-derived peptides with both antihypertensive effects and zinc fortification have good application prospects in the food and medical industries. However, data on ACE inhibition ability and zinc fortification of peptides are limited.

Moreover, the efficiency, bioavailability, safety, physicochemical properties, and stability of bioactive peptides can affect their application in the nutraceutical and medical industries (Singh and Vij, 2018). Peptides with potential toxicity or allergenicity cannot be used in food. Physicochemical properties, including hydrophobicity, hydrophilicity, and isolation points, can influence the coordination of peptides with ACE or zinc ions, thereby affecting the ACE inhibitory activity and stability of peptides in different food systems (Wong et al., 2019). Furthermore, enzymes present in the stomach and intestine can degrade peptides and change their structure, physicochemical properties, and bioactivity (Wang et al., 2021). Hence, the security, stability, and physicochemical properties must be studied before application of new peptides.

Defatted oil palm (*Elaeis guineensis* Jacq.) kernel (DOPK), the main byproduct of the palm oil industry, is a potential plant protein resource. The protein content in DOPK is 14.5–19.5 g/100 g, which is nearly equivalent to that of the soybean, and the amino acid composition profile of defatted oil palm kernel protein (DOPKP) is well-balanced (Chee et al., 2012; Zarei et al., 2016). Furthermore, the annual worldwide yield of DOPK is approximately 460,000 tons (Sathitkowitchai et al., 2022). Globulin is the main fraction of DOPK protein, accounting for 40.10 g/100 g (Tapal et al., 2016). The pre-experiment of this study demonstrated that defatted oil palm kernel globulin hydrolysates (DOPKGH) had both ACE-inhibitory capacity (19.71%) and zinc-chelating ability (11.67 mg/g). Therefore, ACE-inhibitory peptides with a Zn-chelating capacity should be obtained from defatted oil palm kernel globulin (DOPKG). Although anticancer, antihypertensive, and antibacterial peptides have been isolated from DOPKG (Chang et al., 2015; Asri et al., 2020; Zarei et al., 2022), data on oil palm kernel ACE-inhibitory peptides with Zn-chelating abilities are limited. Thus, our first objective was to isolate peptides from DOPKGH with both ACE-inhibitory and Zn-chelating capacities. The inhibitory mechanisms of DOPKGH peptides on ACE and their coordination patterns with zinc ions were studied. Moreover, the physicochemical parameters, stability, and security of the DOPKGH peptides were investigated.

## 2 Materials and methods

### 2.1 Materials

DOPK was provided by the Three Rivers Palm Garden (Haikou, China). Trypsin (1 × 10^4^ U/g, derived from porcine pancreas), papain (3 × 10^4^ U/g), and pepsin (1 × 10^5^ U/g, derived from bovine stomach) were purchased from Kangfukuai Biotechnology Co. (Nanning, China). ACE, 4-(2-Pyridinazo)-resorcinol (PAR), and N-hippuryl-l-histidyl-l-leucine (HHL) were purchased from Sigma (St. Louis, MO, United States). Dithiothreitol, HEPES-KOH buffer, and other chemicals were purchased from Jinyangkeji Co. (Linfen, China).

### 2.2 Preparation of DOPKGH

Globulin was extracted from DOPK following the procedure described by Zheng et al. (2021), with 0.25 mol/L NaCl as the extraction solvent. Then the obtained DOPKG was hydrolyzed with papain to produce peptides of high zinc-chelating capacity. Briefly, DOPKG (2 g) was thoroughly dispersed in 100 mL of dH_2_O, and then adjusted to pH 7.5 with 0.1 mol/L of HCl or 0.1 mol/L of NaOH. Papain (75 mg) was added, and the reaction solution was held at 55°C in a shaking water bath (XBZ-2; Shangyu Co., Hangzhou, China) for 95 min. Subsequently, the reaction solution was heated at 100°C for 8 min. The reaction mixture was cooled and centrifuged at 12,000 *× g* for 12 min. The supernatant was collected and freeze-dried to obtain DOPKGH. In addition, the trinitrobenzenesulfonic acid method was used to determine the hydrolysis degree (Adler-Nissen, 1979).

### 2.3 Isolation of ACE-inhibitory peptides with Zn-chelating ability from DOPKGH

DOPKGH (dissolved in ultrapure water, 1 mg/mL) were passed through a W-45 ultrafiltration membrane with a filter diameter of 0.45 μm (Jieneng Co., Wuxi, China) (Zheng et al., 2021). The filtrate was lyophilized using an LGJ-10N freeze dryer (Keya Instrument Co., Beijing, China). The obtained powder was resolved in ultrapure water (1 mg/mL) and separated with Sephadex G-15 gel chromatography on a column (Ф1.2 × 80 cm). The elution rate was 2.6 mL dH_2_O/min and monitored at 220 nm. The effluent fractions were collected and lyophilized, and their ACE inhibitory activity and Zn-chelating ability were determined (Udechukwu et al., 2018; Zaharuddin et al., 2022). The subfraction with the highest ACE-inhibitory activity was further separated using reverse-phase high performance liquid chromatography (RP-HPLC) on a Kromasil 100-5 C_18_ column (4.6 × 250 mm, 5 μm; Eka Chemicails, Sweden) with deionized water containing 0.1% (v/v) trifluoroacetate as the elution solution A. Moreover, a linear gradient of acetonitrile containing 0.1% TFA (1%–22%, in 15 min) was used as the elution solution B. The flow velocity was maintained at 1.0 mL/min and the monitored wavenumber was 220 nm. The subfractions were separately collected and lyophilized, and the ACE-inhibitory and Zn-chelating activities were determined. The subfraction with the highest capacity was used for amino acid sequence identification.

### 2.4 Determination of ACE-inhibitory activity and inhibition kinetics

ACE inhibitory activity was determined using the procedure described by Zaharuddin et al. (2022). Briefly, ACE (25 mU) was pre-incubated at 37°C for 10 min. Then ACE (75 µL), 225 µL of HHL (8.3 mmol/L), and the peptide solution (75 µL) were mixed and stirred at 75 rpm and 37°C for 60 min. The reaction was stopped by adding 375 µL of HCl (1 mol/L). Afterward 2.1 mL of ethyl acetate was added to extract the hippuric acid produced. The mixture was centrifuged at 14,000 *g* for 150 s. Subsequently, 1 mL of the upper solution (ethyl acetate extraction) was transferred to a glass test tube and heated at 120°C for 32 min. The tube was cooled to 25°C, deionized water (1 mL) was added, and the absorbance was measured at 228 nm. The control group was subjected to the same procedure, but without samples. The ACE inhibition ability of the samples was defined as the percentage of the difference in absorbance at 228 nm between the sample and control as compared to the absorbance at 228 nm of the control. The concentration of peptides required to inhibit half of the ACE activity was defined as the IC_50_.

Moreover, the ACE inhibition kinetics of samples on ACE was analyzed based on the Lineweaver-Burk plot of ACE with the addition of peptides (0–60 μmol/L) identified in DOPKGH, following the same procedures as Urbizo-Reyes et al. (2022). The ACE substrate (HHL) concentration ranged from 0 to 7.60 mmol/L.

### 2.5 Determination of Zn-chelating ability

DOPKGH peptides (350 μg), 0.5 mL zinc sulfate solution (0.25 mmol/L), 0.5 mL DTT (8 mmol/L), 1 mL HEPES-KOH buffer (100 μmol/L), and 8 mL dH_2_O were mixed thoroughly (Li et al., 2023). After stirring at 175 r/min and 37°C for 12 min, the zinc concentration of the reaction solution was measured using the 4-(2-Pyridinazo)-resorcinol method (Udechukwu et al., 2018). The standard regression curve of the zinc concentration (*x*, μg/mL) with absorbance at 500 nm (*y*) was *y* = 0.0901n(*x*) + 0.1012, and R^2^ = 0.9802 (Li et al., 2023). The zinc chelation rate was defined as the reduction of zinc ions in the reaction solution per unit mass of sample (mg/g).

### 2.6 Identification, chemical synthesis, and physicochemical property analysis of the peptide sequence

Amino acid sequence identification was conducted using a Q Exactive hybrid quadrupole orbitrap mass spectrometer (Thermo Fisher, Bremen, Germany) coupled with Peak-Studio-7.5-De-Novo™ software (Bioinformatics Solutions, Inc., Waterloo, Canada) according to the method described by Li et al. (2022). The DOPKGH sequences were verified by matching with the sequences of *Elaeis guineensis* recorded in the Biotechnology Information Database (Bethesda, MD, United States). The Zn-chelating ability of the obtained sequences was measured using the 4-(2-Pyridinazo)-resorcinol method (Udechukwu et al., 2018). Potential antihypertensive effects were predicted using both the BIOPEP and AHTPDB databases (Kumar et al., 2015). The predicted average local confidence (ALC) of antihypertensive peptides should be above 85%, and their vector machine software scores (SVMS) should be higher than zero (Zaharuddin et al., 2022). Chemical synthesis of the selected sequence was performed using the solid-phase synthesis method at Dingxiang Peptide Co. (Shaoxing, China). In addition, the physicochemical characteristics of the screened DOPKGH sequences were analyzed using the AHTPDB database.

### 2.7 Toxicity and allergenicity evaluation

The potential toxicity and allergenicity of the peptides identified in DOPKGH were predicted using ToxinPred (http://www.imtech.res.cn/raghava/toxinpred/) and AlgPred (http://www.imtech.res.cn/raghava/algpred/) databases, respectively (Sudheer et al., 2013).

### 2.8 Molecular docking

The SYBLY-X.2.0.1 Murflex-Docking Tool (Tripos Int. Co., Saint Louis, MO, United States) was used to perform visual molecular simulations of the coordination between the screened DOPKGH peptides and the ACE crystal structure (Li et al., 2022). The ACE structure with the code PDB-108A, downloaded from the Protein Data Bank (http://www.rcsb.org), was used as a molecular docking template. The coordination patterns of DOPKGH peptides with ACE were selected mainly based on the predicted T-scores (the acceptable threshold was 6.0), C-scores, and the number and length of hydrogen bonds. The hydrophobic interactions of the screened DOPKGH peptides with ACE were studied using the LigPlot (Zaharuddin et al., 2022).

### 2.9 Interactions of zinc ions with DOPKGH ACE-inhibitory peptides

#### 2.9.1 Preparation of peptide-Zn complex

In a stirring water bath (175 rpm), the chemically synthesized DOPKGH peptides (200 μg) were dispersed and reacted with 5 mmol/L of ZnSO_4_·7H_2_O (1.4 mL) at pH 6.2°C and 63°C for 55 min (Li et al., 2023). After centrifugation at 4,500 × *g* for 35 min, the supernatant was mixed with four times the volume of anhydrous ethanol and incubated at 25°C for 35 min. The mixed solution was centrifuged at 12,500 × *g* for 8 min, and the precipitate was collected, washed three times with anhydrous ethanol, and lyophilized to obtain DOPKGH peptide-Zn complexes.

#### 2.9.2 Coordination patterns of DOPKGH peptide with zinc ions

The obtained DOPKGH peptide–Zn complexes were thoroughly mixed with dry potassium bromide (1:50, m/m) and pressed into tablets with a thickness of 1–2 mm. The infrared spectrum of the obtained tablets was analyzed using a Fourier-transform infrared spectrometer (660-IR; Varian, United States) with a scanning range of 4000–400 cm^–1^ (Ke et al., 2022). Moreover, the resolution wavenumber was 4 cm^–1^, and the DOPKGH peptides were used for comparison.

#### 2.9.3 ACE inhibition ability of Peptide-Zn complex

The ACE-inhibitory activity of the peptide-Zn complex was determined using the procedure described in Section 2.5. The concentrations of the peptide-Zn complex used were 1 mg∙mL^-1^.

### 2.10 Stability under the simulated gastrointestinal hydrolysis

The simulation intestinal digest mucus (pH 6.80 ± 0.10) was composited of 6 g bile salt, 0.07 g pancreatin, 12.5 g NaHCO_3_, and 200 mL ultrapure water. The simulated gastric digested mucus (pH 2.00 ± 0.10) contained 0.15 mol/L of NaCl and 0.35 mg/mL of pepsin (Wang et al., 2021). Peptides (5 g) were first hydrolyzed with simulated gastric digest mucus (30 mL). The digest was stirred at 135 rpm and 37°C for 80 min. Subsequently, the pH value of the digest was adjusted to pH 6.8 and 50 mL of simulated intestinal digest mucus was added. After stirring at 135 rpm and 37°C for 150 min, the digest was placed at 100°C for 10 min. The ACE inhibitory activity of the DOPKGH peptide was determined using untreated DOPKGH peptides for comparison.

Simultaneously, 5 mg of the DOPKGH peptide-Zn complexes was mixed with 50 mL of simulated gastric digested mucus (pH 2.00 ± 0.10) (Sun et al., 2021). After shaking at 37°C and 135 rpm for 90 min, Na_2_HPO_4_ (0.5 mol/L) was added and the mixture was quickly stirred until the pH of the digest increased to 6.80 ± 0.10. The simulated intestinal mucus digest (50 mL) was then added, and the digest was continuously stirred at 37°C and 135 rpm for 150 min. At 30 min intervals, an aliquot of the digest (0.8 mL) was removed for zinc concentration determination using the 4-(2-Pyridinazo)-resorcinol method (Udechukwu et al., 2018). The stability of DOPKGH peptide-Zn complexes was expressed as the residual zinc concentration after digestion and as a percentage of the zinc concentration before digestion. Zinc gluconate (100 g/mL) and ZnSO_4_ (100 g/mL) were used for comparison.

### 2.11 Antihypertension *in vivo*

After a week of environmental adaptation, thirty-two spontaneous hypertensive rats (SHR, 12 weeks old, 240 ± 15 g body weight, male; Beijing Medicine YaoLi Biotechnology Co., Ltd., China) were randomly divided into eight groups: negative, positive, DOPKGH peptide low-, middle-, and high-groups, and DOPKGH peptide-Zn complex low-, middle-, and high-groups (Kaprasob et al., 2022). Each group consisted of four rats. The rats in the negative control and positive groups were gastrically intubated with NaCl (0.9%) and captopril (10 mg/kg body weight once daily), respectively, while the rats in the high-, middle-, and low-dosage groups were orally administered 150, 100, and 50 mg of DOPKGH peptides (or peptide-Zn complexes)/kg/body weight once daily, respectively. The oral administration continued for 5 weeks. The diastolic and systolic blood pressures of the rats were measured every 7 days using the tail cuff method with a ZL-620-F non-invasive blood pressure apparatus (Anhui Yaokun Biotechnology Co., Ltd., Hefei, China). This experimental study was approved by the Institutional Animal Care and Use Committee of Shanxi Normal University. All animals received human care according to the Guideline Manual for the Care and Use of Laboratory Animals.

### 2.12 Statistical analysis

The results of all tests are expressed as mean ± standard deviation, and each experiment was repeated at least three times. Differences among data were analyzed by one-way analysis of variance coupled with Duncan’s multiple range tests using IBM SPSS Statistics software (Version 16, Chicago, IL, United States). Differences were considered statistically significant at *p* < 0.05.

## 3 Results and discussion

### 3.1 Selection of fractions with high zinc-chelating capacity from DOPKGH

**FIGURE 1:**
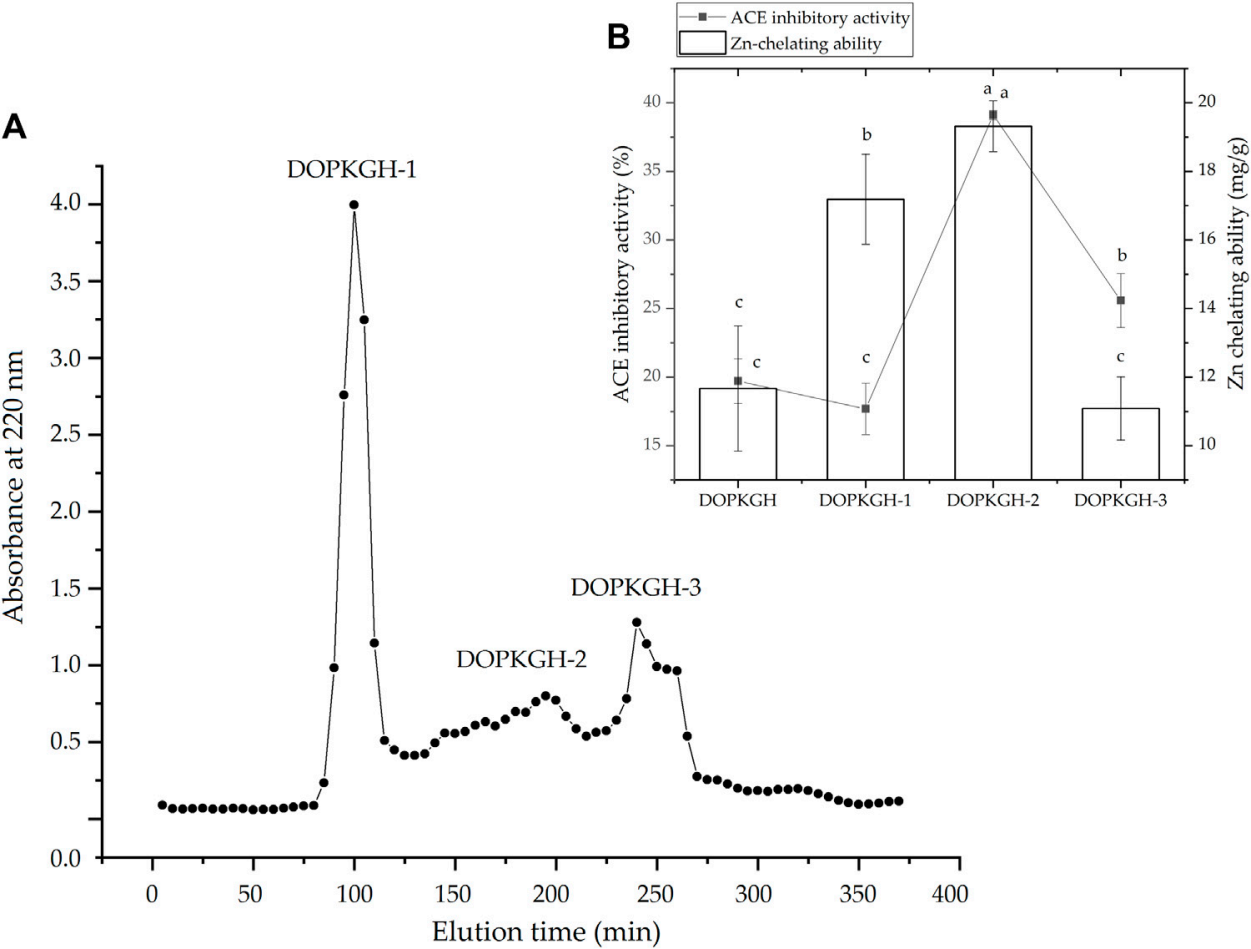
**(A)** The Sephadex G-15 gel chromatographic spectrum of defatted oil palm kernel globulin hydrolysates (DOPKGH), and **(B)** the ACE-inhibitory activity and Zn-chelating ability of the obtained subfractions DOPKGH-1, DOPKGH-2 and DOPKGH-3. Lowercase letters (a–c) on the bars or near the line mean significant difference (*p* < 0.05).

**FIGURE 2:**
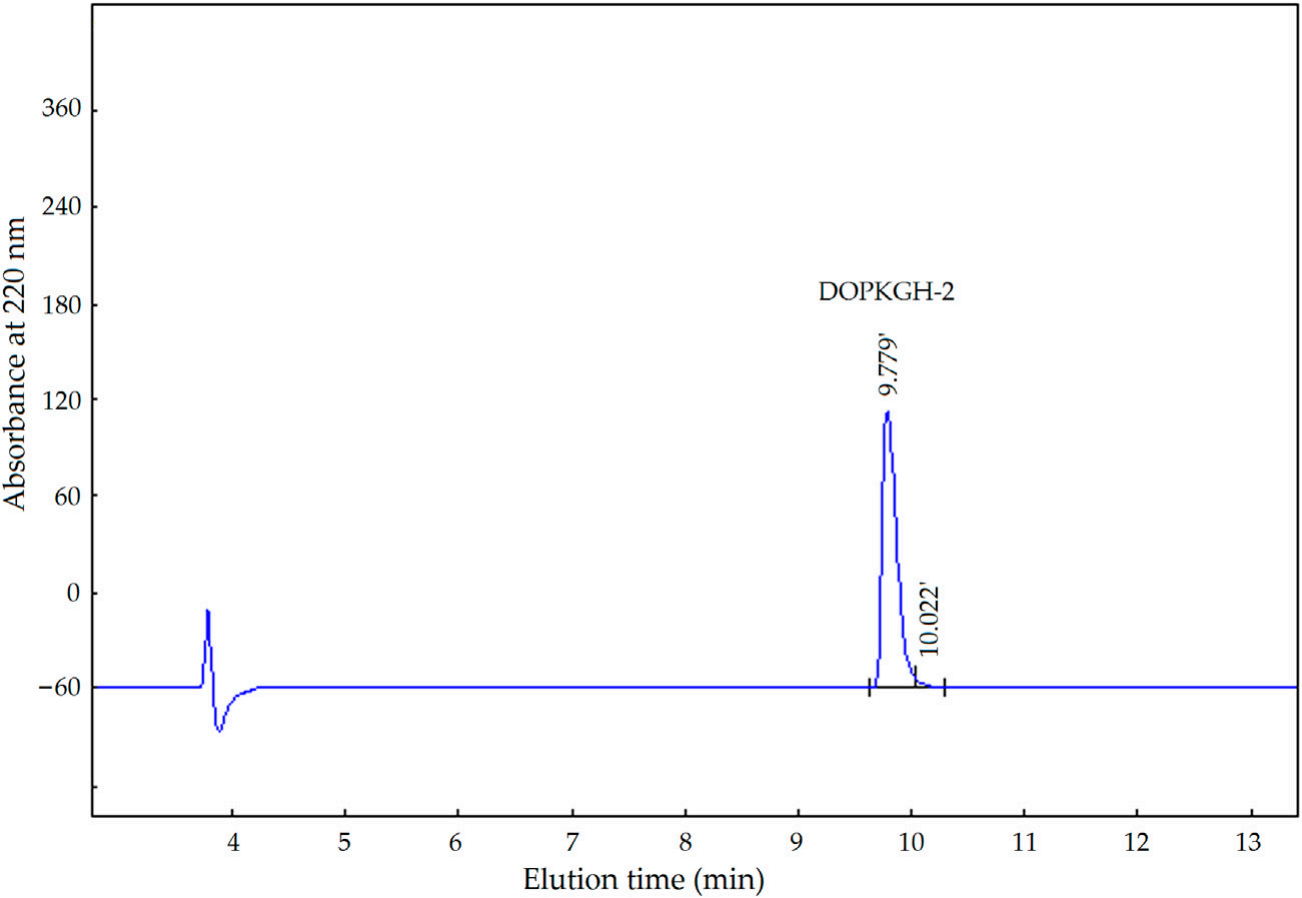
The reverse-phase high performance liquid chromatograpic profiles of subfraction DOPKGH-2.

The degree of hydrolysis of the DOPKG was 31.67%, which is consistent with the results of Tapal et al. (2016). The ACE-inhibitory capacity and zinc-chelating ability of DOPKGH were 19.71% ± 1.62% and 11.67 ± 1.83 mg/g, respectively. The profile in Figure 1A shows that DOPKGH was divided into three major subfractions (DOPKGH-1, DOPKGH-2, and DOPKGH-3) after purification using G-15 gel chromatography. Among them, the DOPKGH-2 exhibited the highest ACE-inhibitory capacity (39.11% ± 0.34%) and zinc-chelating capacity (19.31 ± 0.74 mg/g, Figure 1B). Therefore, it was further purified by reverse-phase high-performance liquid chromatography (RP-HPLC) on an analytical C_18_ column. As shown in Figure 2, only one main subfraction (DOPKGH-2) was obtained, and it was collected and used for amino acid sequence identification.

### 3.2 Identification and characterization of peptides from DOPKGH-2

**FIGURE 3:**
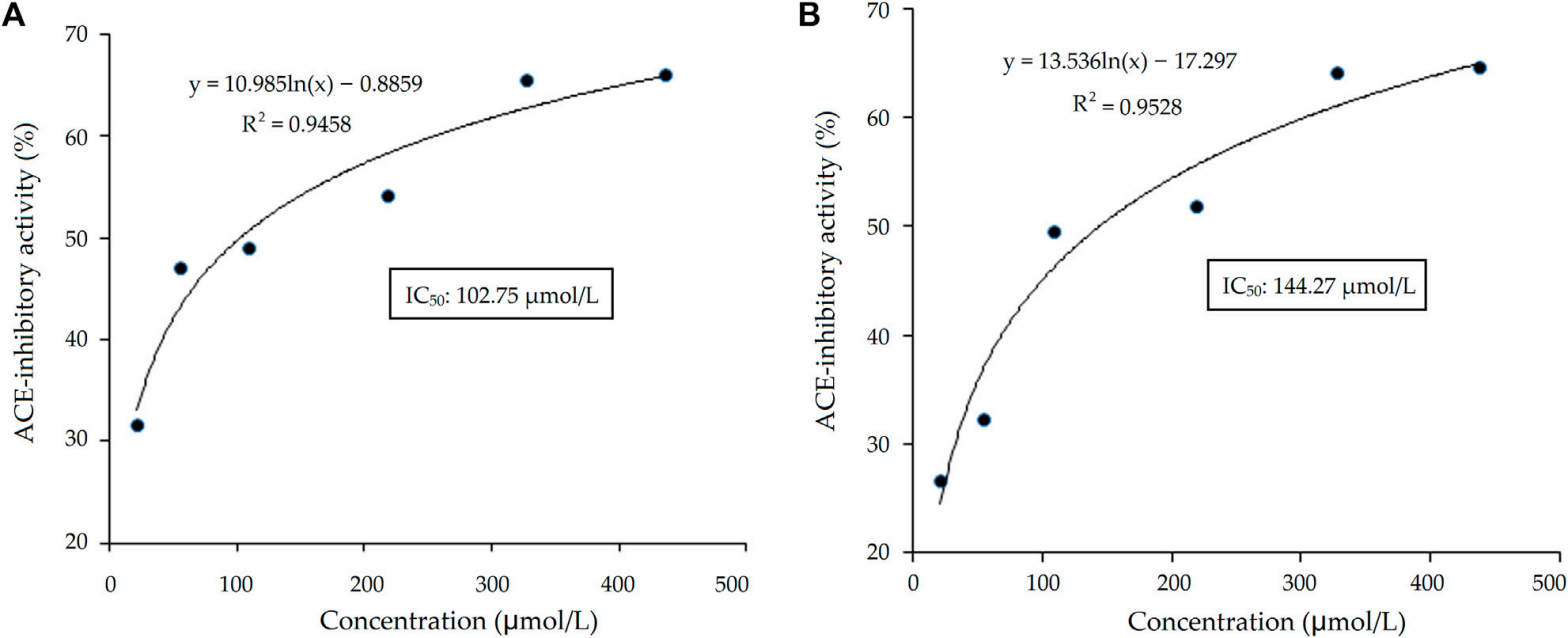
The regression analysis on ACE-inhibitory activity of heptapeptide EVPQAYIP before **(A)** and after the simulated gastrointestinal digestion **(B)**. IC_50_ is the concentration of EVPQAYIP that is needed to inhibit half of ACE activity.

As shown in Table 1, there were five representative oligopeptides including Gln-Arg-Leu-Asp-Arg-Cys-Lys (QRLERCK), Leu-Leu-Leu-Gly-Val-Ala-Asn-Tyr-Arg (LLLGVANYR), Arg-Ala-Asp-Val-Phe-Asn-Pro-Arg (RADVFNPR), Arg-Val-Ile-Lys-Tyr-Asn-Gly-Gly-Gly-Ser-Gly (RVIKYNGGGSG), and Glu-Val-Pro-Gln-Ala-Tyr-Ile-Pro (EVPQAYIP) identified in DOPKGH-2-C based on the results of UPLC-ESI-MS/MS analysis. The *in silico* screening results showed that only EVPQAYIP had both potential ACE-inhibition and antihypertensive effects. As shown in Figure 3A, the relationship between synthesized EVPQAYIP ACE-inhibition ability (*y*) with its concentration (*x*) was *y* = 10.985, ln(*x*) ‒ 0.8859, and R^2^ = 0.9458, from which its IC_50_ value was calculated as 102.75 μmol/L. Moreover, the ACE-inhibition ability of EVPQAYIP was higher than that of peptides with similar mass such as GNPLSP derived from *Arthrospira platensis* (IC_50_: 264.24 μmol/L; Wang et al., 2021) and PHQPLPP identified in rubing cheese (IC_50_: 0.821 mg/mL, Wei et al., 2022) (*p* < 0.05), suggesting that EVPQAYIP is a relatively effective ACE-inhibitor.

**TABLE 1:** Amino acid sequences, Zn-chelating capacity, and *in silico* prediction on physicochemical properties, toxicity and allergenicity of peptides identified in defatted oil palm kernel globulin hydrolysates.

Peptide sequence	QRLERCK	LLLGVANYR	RADVFNPR	RVIKYNGGGSG	EVPQAYIP
Molecular weight (Da)	932.19	1018.33	974.18	1107.39	916.14
Matched sequence in Elaeis guineensis ^a^	K.QRLERCK.Q	K.LLLGVANYR.V	R.RADVFNPR.G	D.RVIKYNGGGSG.G	R.EVPQAYIP.G
SVMS ^b^	‒0.78	‒0.99	‒0.71	‒0.27	2.03
Antihypertension prediction	Non-AHT	Non-AHT	Non-AHT	Non-AHT	AHT
ACE inhibition capacity (IC_50_: μmol/L)	ND ^c^	ND	ND	ND	102.75
Zinc chelating capacity (mg/g)	17.30 ± 1.46^g^	3.00 ± 0.12^i^	4.46 ± 0.22^i^	2.48 ± 0.09^i^	11.69 ± 0.38^h^
Hydrophobic amino acid content (%)	12.50	55.56	50.00	18.18	62.50
Hydrophobicity	‒0.77	0.02	‒0.44	‒0.26	0.01
Amphiphilicity	1.58	0.83	0.61	0.49	0.95
Hydrophilicity	1.34	‒0.72	0.59	‒0.16	‒0.36
Isoelectric point	9.55	9.10	9.95	10.11	4.00
Toxicity ^d^	Non-Toxin	Non-Toxin	Non-Toxin	Non-Toxin	Non-Toxin
Allergenicity	No	No	No	No	No

^a^ From National Center for Biotechnology Information (NCBI).

^b^ Physicochemical properties including hydrophobicity, amphiphilicity, hydrophilicity and isoelectric point were *in silico* predicted using the AHTPDB database (http://crdd.osdd.net/raghava/ahtpdb/).

^c^ND: not measured. Different lowercase letters (g,i,h) in the same line means significant difference (*p* < 0.05).

^d^ The potential toxicity and allergenicity were predicted using the database ToxinPred (www.imtech.res.in/raghava/toxinpred/) and AlgPred (www.imtech.res.in/raghava/algpred/), respectively.

It has been demonstrated that hydrophobic amino acids in *C*-terminal tripeptide, especially Phe and Trp (aromatic amino acids), Leu, Ile, Val (branched amino acids), and Pro residues, play a crucial role in the inhibitory effect of peptides on ACE, as these hydrophobic amino acids have a relatively high binding affinity for the active sites of ACE (Chen et al., 2022). Moreover, Lys or Arg residues in *C*-terminal tripeptide are instrumental in the ACE-inhibitory ability of peptides (Urbizo-Reyes et al., 2022). Additionally, previous studies have also found that the Val residue near the *N*-terminal is helpful for the coordination of peptides with ACE (Zaharuddin et al., 2022). The Val residue at the *N*-terminal of VSWNVLQEP, identified in King Bolete mushrooms, was demonstrated to have strong binding energy (−9.1 kcal/mol) with ACE (Kaprasob et al., 2022). Therefore, the hydrophobic amino acids, especially Pro, Ile, Val, and Tyr residues, in the *C*-terminal tripeptide or near the *N*-terminal, mainly contributed to the high inhibitory capacity of EVPQAYIP against ACE.

**FIGURE 4:**
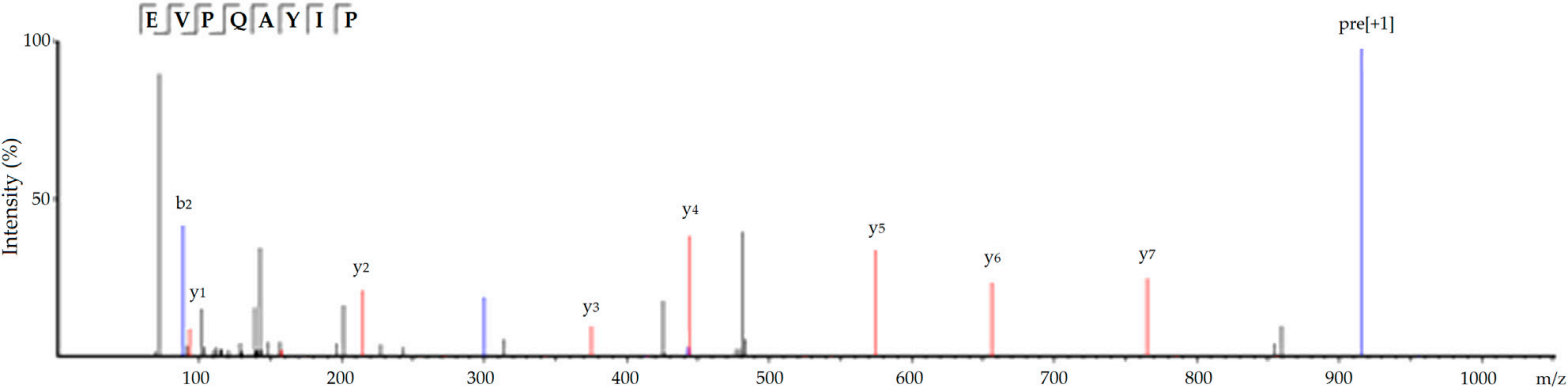
The ESI-MS/MS spectrum of peptide EVVPQAYIP identified in defatted oil palm kernel globulin hydrolysates.

Moreover, EVPQAYIP exhibited considerable Zn-chelating ability (11.69 ± 0.38 mg/g, Table 1). A study on the structure-activity relationship of peptide-Zn chelates showed that peptides with terminal amino and/or carbonyl groups had a high chelating ability with Zn ions (Wang et al., 2020). The nitrogen atoms of the second and third amide residues at *N*-terminal can take part in the coordination with zinc ions (Udechukwu et al., 2018). Glu or Asp with γ-carboxyl group can increase the negative polarity of peptides and improve the binding force between zinc ions and peptides. Additionally, Pro residues can be used as a negative bridge ligand for zinc ions (Pina and Roque, 2009; Lin et al., 2023). Hence, amino acid residues, especially Pro and Glu, were predominantly responsible for the high binding affinity of EVPQAYIP for zinc ions. Figure 4 shows the ESI-MS/MS spectrum of EVPQAYIP.

### 3.3 Physicochemical characterization *in silico*

As shown in Table 1, EVPQAYIP had a high content of hydrophobic amino acids (62.50%) and relatively high hydrophobicity (0.01), which was another reason for its high ACE-inhibitory capacity. Hydrophobic residues can increase the binding affinity of peptides to active sites of ACE (Pina and Roque, 2009). Moreover, QRLERCK exhibited the highest hydrophilicity (1.34), corresponding to its high Zn-chelating ability (17.30 ± 1.46 mg/g), because hydrophilic groups of peptides have more polar charge for binding with zinc ions (Wei et al., 2022). EVPQAYIP also showed considerable binding affinity with zinc ions (11.69 ± 0.38 mg/g), which was mainly attributed to the Glu residue in *N*-terminal. A previous study found that the-carboxyl group is a good chelating site for Zn ions (Sun et al., 2021).

Additionally, the isoelectric point (pI) of EVPQAYIP was 4.00. Peptides should avoid being used in solutions with pH values near their isoelectric points. At isoelectric points, the polarity and solubility of peptides dramatically decease, leading to an adverse effect on the coordination of peptides with ACE or zinc ions (Zheng et al., 2021).

### 3.4 Security prediction *in silico*

As shown in Table 1, the *in silico* prediction results using the ToxinPred database showed that QRLERCK, LLLGVANYR, RADVFNPR, RVIKYNGGGSG, and EVPQAYIP were peptides without any toxicity. Furthermore, these peptides did not match any allergenic peptide sequences recorded in the AlgPred database. Additionally, short oligopeptides are less likely to have complete epitopes than those with larger masses (Piovesana et al., 2018). These results indicate that these four peptides have no potential allergenicity. However, further security studies, including cellular tests and *in vivo* assays, are required.

### 3.5 Inhibition mechanisms of EVPQAYIP on ACE

#### 3.5.1 Molecular docking

**FIGURE 5:**
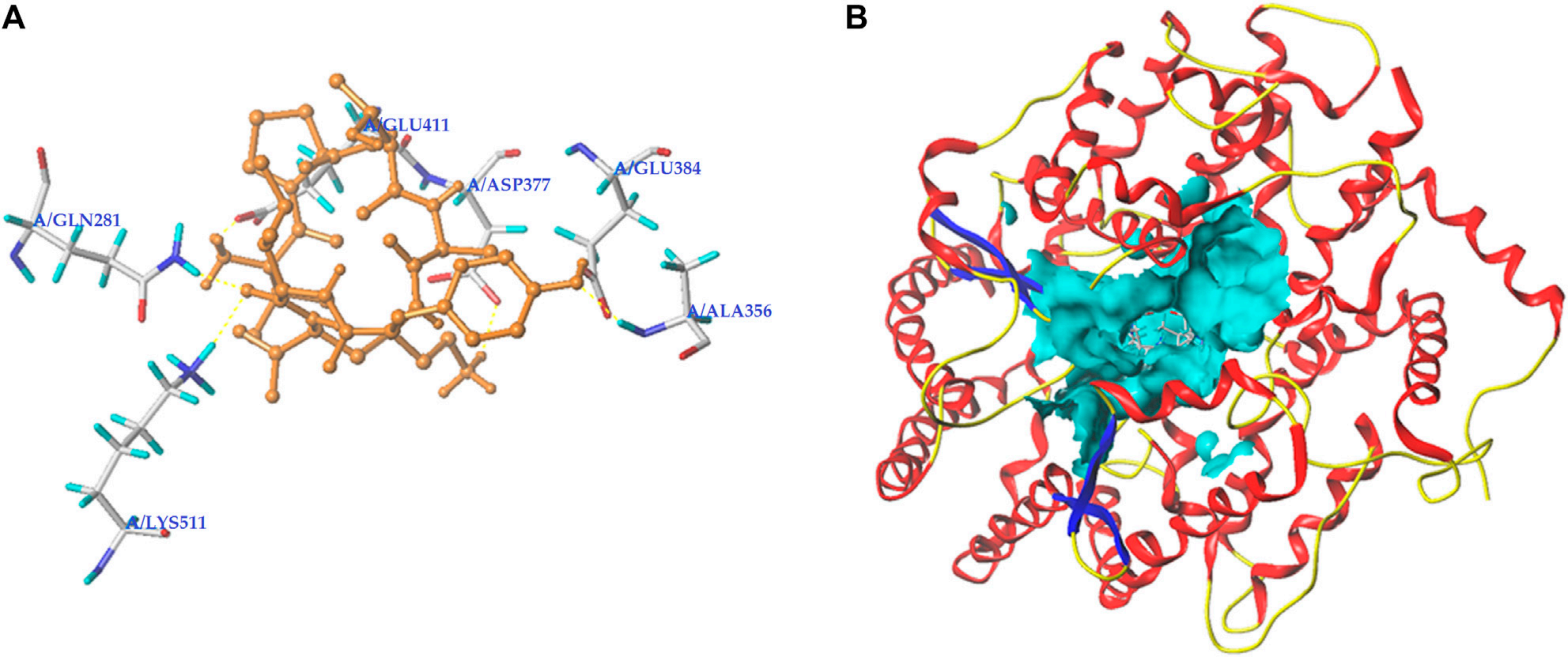
Three-dimensional images for the partial overview **(A)** and the general overview **(B)** of the best-ranked docking mode between EVPQAYIP and ACE (PDB: 1O8A).

Peptides, which can bind with the active sites belonging to S1, S2, or S′ pocket of ACE, have been shown to be efficient competitive ACE inhibitors (Zarei et al., 2022). Figures 5A,B depict the best docking modes of EVPQAYIP and ACE (PDB:1O8A) in local and general views, respectively. As shown in Figure 5A, EVPQAYIP and the six active sites of ACE (Asp377, Glu411, Glu384, Ala356, Gln281, and Lys511) were linked by seven short hydrogen bonds. Of these, Glu384 belongs to the S1 pocket of ACE, whereas Gln281 and Lys511 belong to the S2 pocket (Zheng et al., 2021), suggesting that EVPQAYIP competitively binds to the substrate in the central S1 and S2 pockets of ACE. Additionally, EVPQAYIP interacted with 16 active sites of ACE, including Tyr523, which belongs to the S1 pocket of ACE (Table 2), via hydrophobic interactions.

**TABLE 2:** Interactions between EVPQAYIK and the active sites of ACE analysed using molecular docking.

Peptides	Total score	Consistency score	Interaction mode	Active sites, and the length of hydrogen bonds between these sites and EVPQAYIK
EVPQAYIK	14.74	4	Hydrogen bond	Asp377: 1.99Å; Glu411: 1.88Å; Glu384: 1.88Å; Ala356: 1.88Å; Gln281: 1.91Å; Lys511: 2.50Å
			Hydrophobic interaction	Leu139, Asn66, Ser517, Trp357, Phe391, Tyr360, Ser355, Glu411, Arg522, His383, Tyr523, Val351, Phe512, His353, Val518, Asn70

Hydrogen bonds are instrumental in the coordination of peptides with ACE (Li et al., 2022). The results in Figure 5A and Table 2 show that there were seven hydrogen bonds between EVPQAYIP and ACE with short distances (1.88–2.50 Å). In addition, the total score (T-score) of the EVPQAYIP with ACE (14.74, Table 2) was higher than the acceptable threshold (6.0) (Zheng et al., 2021). The T-score reflects the affinity of peptides for ACE, which is dependent on hydrogen bonds, van der Waals forces, and hydrophobic interactions (Zarei et al., 2022). These results demonstrate that the affinity of EVPQAYIP for ACE was strong, corresponding to its high ACE-inhibitory activity (IC_50_: 102.75 μmol/L).

Moreover, EVPQAYIP can bind with Glu411, which belongs to the zinc tetrahedral coordination of ACE through short hydrogen bonds (1.88 Å) and hydrophobic interactions, respectively (Figure 5A; Table 2). Zinc tetrahedral coordination (the zinc ion coordinates with residues His387, Glu411, and His383) is located in the catalytic center of ACE and plays an important role in the catalytic action of ACE (Kumar et al., 2015). The interactions between EVPQAYIP and Glu411 indicate that EVPQAYIP could inhibit ACE by affecting the zinc tetrahedral coordination. Oligopeptides, including RSRGVFF, KYPHVF, and HPVTGL, identified in *Lepidium* and *symbiot* proteins, could also affect zinc tetrahedral coordination by forming hydrogen bonds with Glu411 of ACE, and showed good ACE-inhibitory activities (Li et al., 2022; Lin et al., 2023).

#### 3.5.2 Inhibition kinetics of EVPQAYIP on ACE

**FIGURE 6:**
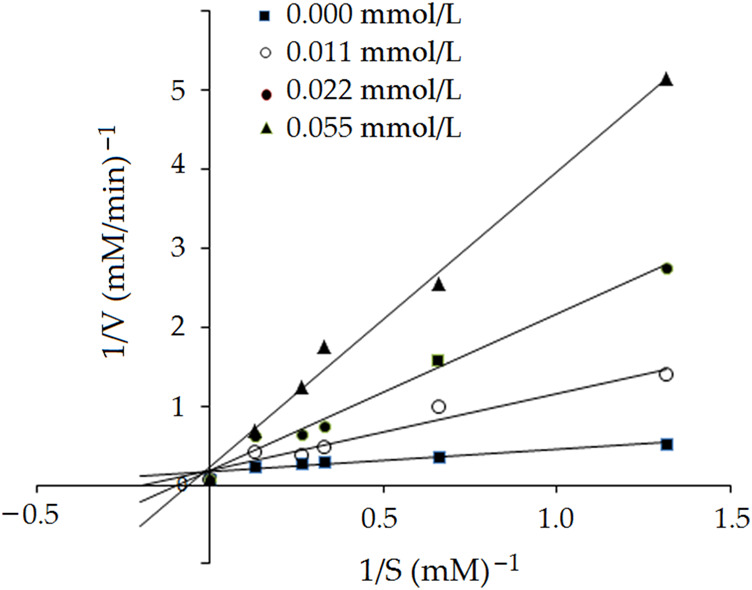
Lineweaver–Burk plots of the ACE inhibition for peptide EVPQAYIP.

Lineweaver-Burk plots of ACE against different concentrations of HHL with the peptide EVPQAYIP are shown in Figure 6. The kinetic constants demonstrated that *K*_*m*_ increased as the concentration of EVPQAYIP increased, whereas V_max_ (maximum velocity) of the reaction was constant. These results suggest that EVPQAYIP is a competitive inhibitor of ACE. This result is consistent with the molecular docking results (Figure 5; Table 2). EVPQAYIP could bind to the active residues Glu384, Gln281, and Lys511 in the S1 and S2 pockets of ACE, thereby exhibiting a competitive inhibition model for ACE.

#### 3.5.3 Coordination patterns between EVPQAYIP and zinc ions

**FIGURE 7:**
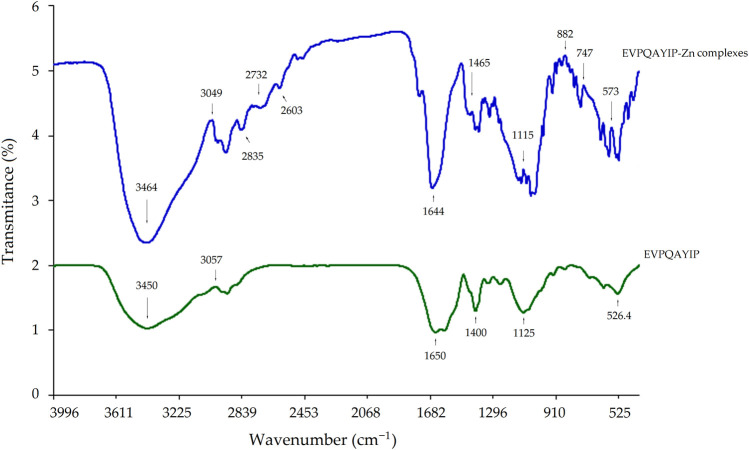
Fourier-transformed infrared spectra of EVPQAYIP-Zn chelate and pure EVPQAYIP.

The coordination patterns of EVPQAYIP with zinc ions were further studied by FT-IR spectroscopy, and the results are shown in Figure 7. Significant differences were observed between the FTIR spectra of the EVPQAYIP-Zn complexes and EVPQAYIP. The peak at 3450 cm^-1^ in the infrared spectrum of EVPQAYIP indicated the deformation of–N–H (Wang et al., 2020), whereas in the infrared spectrum of the EVPQAYIP-Zn complexes, this peak appeared at 3464 cm^-1^. Moreover, the peak at 1400 cm^-1^ in the spectrum of EVPQAYIP, which represents the stretching vibration of the–C–N bond, shifted to 1465 cm^-1^ after Zn chelation (Lin et al., 2023). Additionally, new peaks appeared at 573, 747, and 882 cm^-1^ in the FT-IR spectrum of the EVPQAYIP-Zn chelate, corresponding to stretching of the amide IV band. These results demonstrate that the amino and amide bonds of EVPQAYIP were chelated with zinc ions (Sun et al., 2021). Additionally, the new peaks appeared at 2603, 2732 and 2835 cm^-1^ (all representative of the deformation vibration of carboxyl group) suggested the formation of–COO–Zn (Kaprasob et al., 2022). Therefore, the-carboxyl group in the Glu residue, α-amino group in the Pro residue, and the amide bonds of EVPQAYIP can chelate zinc ions.

### 3.6 Stability against gastrointestinal digestion

Bioactive peptides should be able to resist gastrointestinal digestion to enter the blood and exhibit health benefits (Magouz et al., 2023). As shown in Figure 3B, the relationship between the ACE inhibition ability (*y*) of EVPQAYIP and its concentration (*x*) conformed to the following regression equation: *y* = 13.536ln(*x*) ‒ 17.297, R^2^ = 0.9528. Therefore, the IC_50_ value of EVPQAYIP against ACE was 144.27 μmol/L after gastrointestinal digestion, which was higher than that of the untreated EVPQAYIP (102.75 μmol/L, Figure 3A), evidencing the inhibition ability of EVPQAYIP on ACE was reduced after gastrointestinal hydrolysis. However, EVPQAYIP retained 71.23% of its ACE-inhibitory activity against gastrointestinal digestion.

**FIGURE 8:**
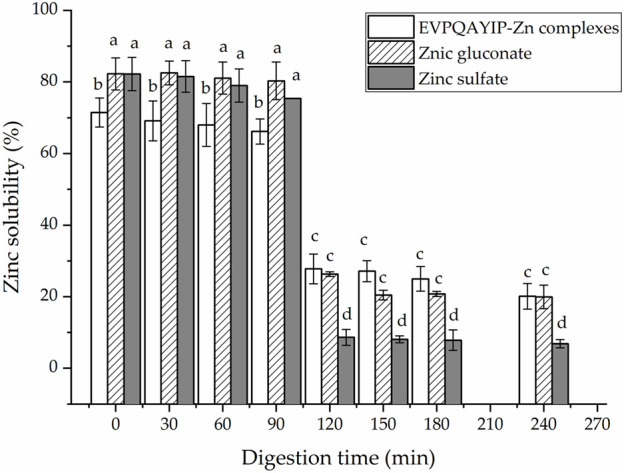
Zinc solubility of EVPQAYIP-Zn complexes, ZnSO_4_ and zinc gluconate against simulated gastrointestinal digestion. Different lowercase letters (a–d) on the bars indicate significant differences (*p* < 0.05).

Moreover, as shown in Figure 8, during digestion with gastric digestive mucus (0–90 min), the EVPQAYIP-Zn complexes exhibited relatively stable Zn-solubility (66.15%–71.47%). However, Zn solubility dramatically decreased during digestion with intestinal digestive mucus (10–240 min) (*p* < 0.05). ZnSO_4_ and zinc gluconate exhibit similar trends. The main reason for this was that the pH of the digestive fluid increased dramatically from 2.0 to 7.0. Most Zn ions in the digestive fluid form insoluble Zn salts as the pH increases (Wang et al., 2020). From 120 to 240 min, both the EVPQAYIP-Zn complexes and zinc gluconate showed higher zinc solubility than that of zinc sulfate (*p* < 0.05), indicating that EVPQAYIP can improve zinc stability in the intestine. In peptide-zinc complexes, zinc ions are generally located inside the peptide chain structure, protecting zinc ions from pH interference (Sun et al., 2021).

It has been shown that enzymes present in gastric or intestinal tissues can cleave polypeptides, especially peptide chains with Phe, Tyr, Lys, and Arg residues, resulting in a decrease in the bioactivity of peptides (Lee and Hur, 2017). Although EVPQAYIP contains a Tyr residue, it exhibits relatively stable ACE-inhibitory activity and Zn-chelating capacity under gastrointestinal hydrolysis, which is probably attributed to the Pro residues (with a rigid ring structure) present in EVPQAYIP (Zhang et al., 2022). Peptides containing Pro residues, such as KPVPR, QPHQPLPP, and INPPSTTN, identified in canary seed and kenaf proteins, respectively, are stable during gastrointestinal digestion (Urbizo-Reyes U et al., 2022; Zaharuddin et al., 2022). However, further studies are needed to investigate the effects of gastrointestinal hydrolysis on the structures of EVPQAYIP and EVPQAYIP-Zn complexes.

### 3.7 Antihypertension *in vivo* of EVPQAYIP and EVPQAYIP-Zn complexes

**FIGURE 9:**
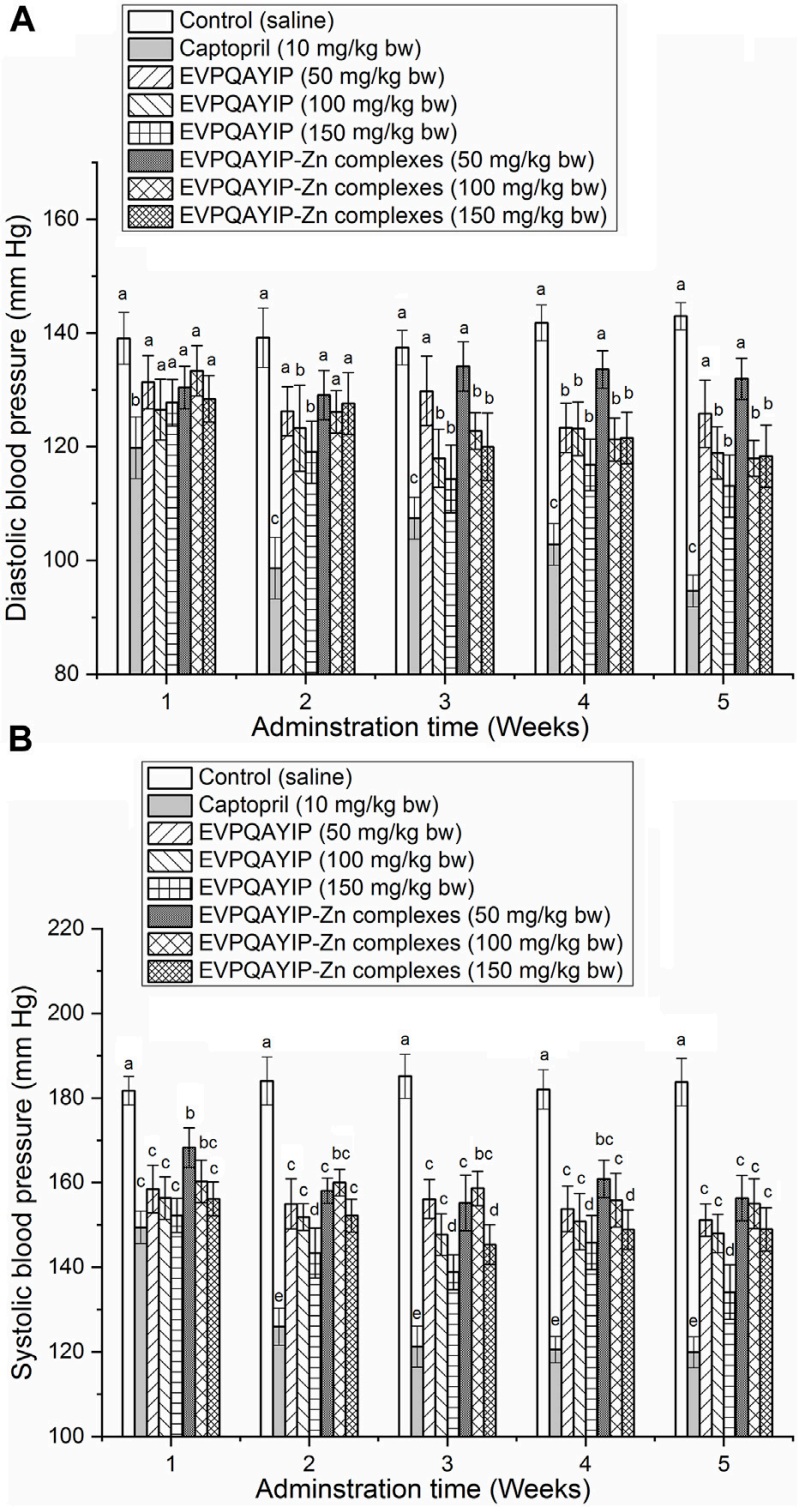
**(A)** Efficiency of EVPQAYIP and EVPQAYIP-Zn complexes on the diastolic blood pressure of spontaneously hypertensive rats. Different small letters (a–c) means significant difference (*p* < 0.05). **(B)** Efficiency of EVPQAYIP and EVPQAYIP-Zn complexes on the systolic blood pressure of spontaneously hypertensive rats. Different small letters (a–e) means significant difference (*p* < 0.05).

The effects of oral administration of EVPQAYIP and EVPQAYIP-Zn on diastolic blood pressure (DBP) and systolic blood pressure (SBP) of spontaneously hypertensive rats (SHR) are shown in Figures 9A,B, respectively. As shown in Figure 9B, both EVPQAYIP and EVPQAYIP-Zn complexes (50–150 mg/kg body weight) significantly decreased the systolic blood pressure of SHR from the first week of oral administration (*p* < 0.05). From the second week, oral administration of EVPQAYIP (100–150 mg/kg body weight) remarkably reduced the diastolic blood pressure of SHR (*p* < 0.05) (Figure 9A). EVPQAYIP-Zn complexes at 100–150 mg/kg body weight significantly lowered the diastolic blood pressure of SHR from the third week (*p* < 0.05) (Figure 9A). These results suggest that both EVPQAYIP and EVPQAYIP-Zn complexes have potential antihypertensive effects *in vivo,* although their efficiency was lower than that of captopril (*p* < 0.05). Moreover, EVPQAYIP and EVPQAYIP-Zn reduced the diastolic and systolic blood pressure of SHR in a dose-dependent manner. Additionally, at the same dose, the DBP (or SBP) of SHR orally administered EVPQAYIP was not significantly different from that of SHR orally administered EVPQAYIP-Zn complexes (*p* > 0.05), suggesting that chelation with zinc ions did not affect the potential antihypertensive effect of EVPQAYIP.

## 4 Conclusion

A competitive ACE inhibitor, EVPQAYIP, with Zn-chelating ability (11.69 mg/g) was identified in DOPKGH. The ACE inhibition modes of EVPQAYIP included the following: 1) EVPQAYIP could bind to six active residues in ACE, including Glu384 (belonging to the S1 pocket of ACE), Gln281, and Lys511 (belonging to the S2 pocket) through seven short hydrogen bonds; 2) EVPQAYIP could bind to 16 active sites through hydrophobic interactions; and 3) EVPQAYIP affected zinc tetrahedral coordination in ACE by binding with Glu411. Moreover, EVPQAYIP retained 71.23% of its ACE inhibitory activity after gastrointestinal hydrolysis. EVPQAYIP could enhance zinc stability in the intestine and exert a potential antihypertensive effect in SHR. These results suggest that EVPQAYIP, isolated from OPKG, can be exploited as an ingredient for antihypertensive agents or zinc fortification. However, further studies are necessary to determine the antihypertensive and zinc supplementation effects.

## Data Availability

The datasets presented in this study can be found in online repositories. The names of the repository/repositories and accession number(s) can be found in the article/Supplementary Material.
